# Revitalizing the AZT Through of the Selenium: An Approach in Human Triple Negative Breast Cancer Cell Line

**DOI:** 10.3389/fonc.2018.00525

**Published:** 2018-11-14

**Authors:** Mônica Silveira Wagner, Eduarda Schultze, Thais Larre Oliveira, Priscila Marques Moura de Leon, Helena Strelow Thurow, Vinicius Farias Campos, Isabel Oliveira, Diego de Souza, Oscar Endrigo Dorneles Rodrigues, Tiago Collares, Fabiana Kömmling Seixas

**Affiliations:** ^1^Programa de Pós-Graduação em Biotecnologia, Grupo de Pesquisa em Oncologia Celular e Molecular, Biotecnologia/Centro de Desenvolvimento Tecnológico, Universidade Federal de Pelotas, Pelotas, Brazil; ^2^Departamento de Fisiologia e Farmacologia, Universidade Federal de Pelotas, Pelotas, Brazil; ^3^LabSelen-NanoBio - Universidade de Federal de Santa Maria, Santa Maria, Brazil

**Keywords:** AZT, selenium, breast cancer, triple negative, apoptosis induction, anticancer agents

## Abstract

Triple-negative breast cancer represents about 15% of all cases of breast cancer, and still represents a therapeutic challenge. 3′-Azido-3′-deoxythymidine (AZT) is a nucleoside reverse transcriptase inhibitor with antitumor activity. Chalcogenides compounds, such as selenium, are very important intermediates applied in organic synthesis. Our objective was to investigate the effect and the underlying cell death mechanisms of AZT and its derivatives, in human breast cancer cell lines. The inhibitory effect of AZT and derivatives (1072, 1073, and 1079) was determined by MTT assay (0.1, 1, 10, 50, and 100 μM for concentrations and times 4, 24, 48, and 72 h) and Live/Dead in tumor cell lines MCF-7, MDA-MB 231 and also in non-tumor cell line CHO. Gene expression profiles related to apoptosis were investigated by qRT-PCR and induction of apoptosis was investigated by flow cytometry. MTT and Live/Dead assays showed that AZT derivatives decreased the rate of cell proliferation at concentrations of 50 and 100 μM in tumor cell lines MCF-7 and MDA-MB 231 while the commercial AZT presented a low antitumoral potential in all strains tested. In flow cytometry analysis we demonstrated that derivatives of AZT induced apoptosis, with an increase in both initial and late stages in both tumor cell lines evaluated, especially in MDA-MB 231. Our data show that the AZT derivative 1072 increased the expression of transcripts of the genes caspase 3 and 8 in MDA-MB 231 cell line when compared to control, suggesting that the extrinsic pathway of apoptosis was activated. In conclusion, derivatives of AZT, especially 1072, induce cytotoxicity *in vitro* in the triple negative breast cancer cell line through activation of the extrinsic pathway of apoptosis. These compounds containing selenium in its formulation are potential therapeutic agents for breast cancer.

## Introduction

Breast cancer is the most frequently diagnosed cancer and the main cause of cancer-related death among females worldwide, with an estimative of more than one million cases per year (1). A set of molecular alterations complex is involved in tumorigenesis and tumor evolution, such changes may confer to tumor cells greater proliferative potential, evasion of apoptosis, sustained vascularization, and ability to tissue invasion and metastasis (2). Triple-negative breast cancer is characterized by the lack of expression of estrogen receptors (ER), progesterone receptors (PgR), and the HER-2 gene (3). This type of tumor comprises about 15% of all cases of breast cancer and still represents a therapeutic challenge, due to its poor prognosis and no standard treatment available to date (4).

Several nucleoside analogs have showed important anti-viral and anti-tumoral activities (5). 3′-Azido-3′-deoxythymidine (AZT) is a nucleoside analog used in the treatment of acquired immunodeficiency syndrome (AIDS) due to its antiretroviral activity, however it was firstly developed as an anti-cancer agent (6). Anti-neoplasic potential of AZT has been shown for several tumor cell lines, including those derived from colon (7), breast (8), bladder (9, 10), and esophageal (11) cancers.

AZT effects in the inhibition of cancer cell growth likely involve several biological mechanisms. AZT incorporates into DNA during replication and blocks chain elongation. It has also been described as a telomerase inhibitor (12) and a substrate of thymidine kinase (TK), an enzyme responsible for thymidine phosphorylation [9]. The potential of AZT as an antiproliferative agent is highlighted by the increased thymidine synthesis in tumor cells and mitochondrial toxicity associated with prolonged exposition to this drug (13). However, due to several drawbacks of AZT therapy, such as bone marrow toxicity, myopathy, low blood brain barrier uptake and short half-life in plasma, efforts have been directed to the development and characterization of new AZT-derivated compounds (14, 15).

Chalcogenides compounds, such as selenium (Se) and tellurium (Te), are very important intermediates and reagents used in organic synthesis. These compounds have been associated with improvement of antioxidant and antitumoral effects of several molecules (16, 17). Selenium is an essential element involved in many cellular function including antioxidant pathways, and its effects on cell proliferation has been investigated (18). Sufficient intakes of this trace element have been associated with prevention of many types of cancer, mainly prostate and colorectal. Chemical derivatives of Se have been developed and their potential in cancer chemotherapy have been demonstrated (17, 19).

Therefore, the aim of our study was to investigate the cytotoxic effect of AZT and seleno-AZT derivatives on human breast cancer cell lines and characterize the underlying cell death-related mechanisms.

## Materials and methods

### Chemical

**Figure 1:**
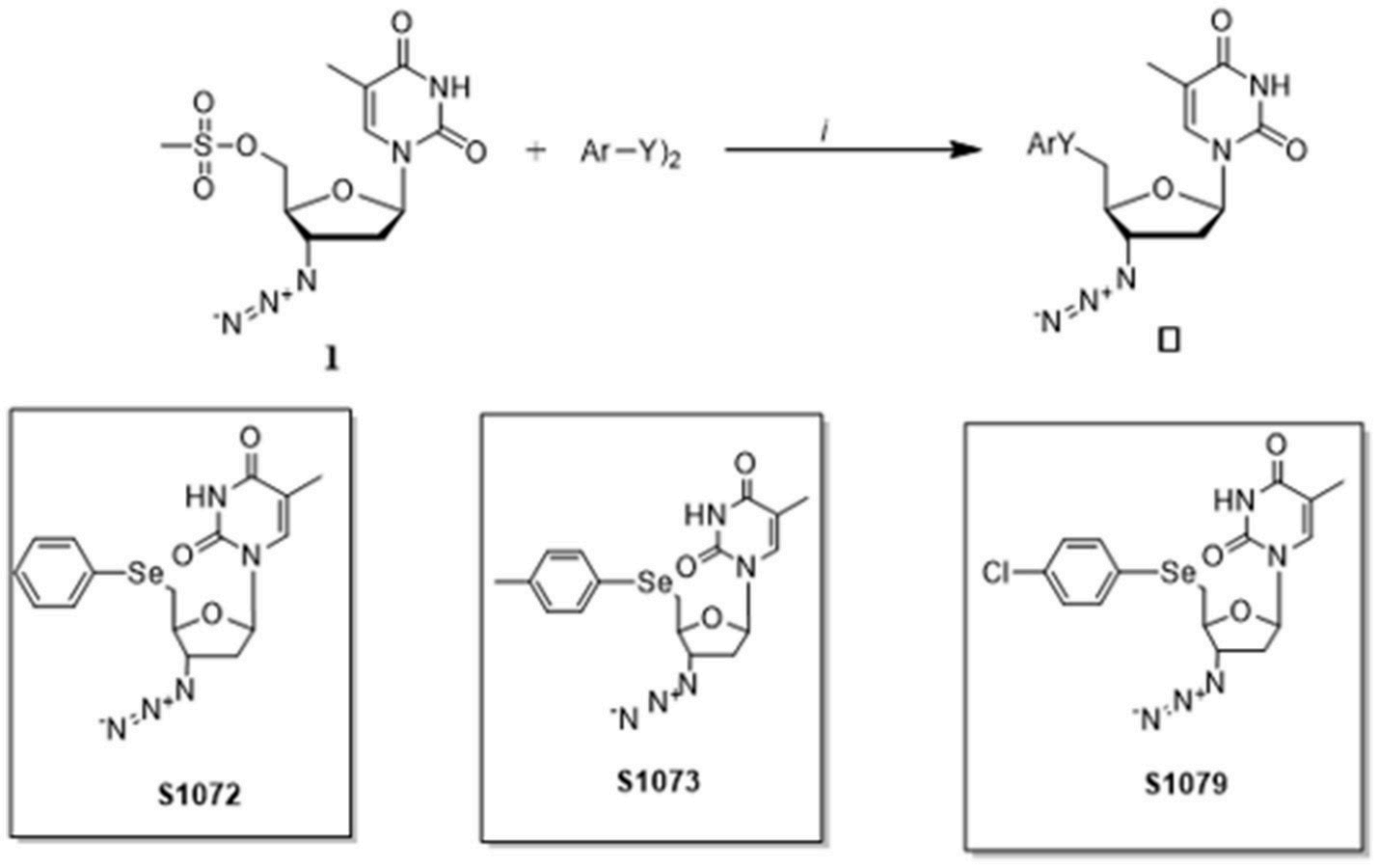
Synthesis of seleno-AZT derivatives.

The seleno-AZT derivatives were synthesized in the Department of Chemistry, University of Santa Maria as previously reported (17). Briefly, in a flask under argon atmosphere diaryl dichalcogenide (0.6 mmol) solubilized in THF (3 mL) and ethanol (2 mL), was treated with NaBH4 (1.0 Equation) and the reaction was stirred until a colorless appearance. Further, the zidovudine-mesylate 1 (1 mmol) dissolved in THF (3 mL) was added dropwise to the reaction flask and stirred at room temperature for 6 h, affording the respective seleno-AZT derivatives S1072, S1073, and S1079 (Figure 1).

### Cell culture

MCF-7 (moderately invasive) and MDA-MB 231 (highly metastatic), human mammary adenocarcinoma cell lines, and CHO, a non-tumor cell line derived from the ovary of the Chinese hamster, were obtained from the Rio de Janeiro Cell Bank (PABCAM, Federal University of Rio de Janeiro, RJ, Brazil). MCF-7, MDA-MB 231, and CHO cell cultures were maintained in RPMI1640, supplemented with 20% of fetal bovine serum (FBS), Leibovitz's supplemented with 10% FBS and Dulbecco's modified Eagle's medium (DMEM) supplemented with 10% FBS, purchased from Vitrocell Embriolife (Campinas, Brazil) and Gibco (New York, USA), respectively. Cells were grown at 37°C in an atmosphere of 95% humidified air and 5% CO_2_. The experiments were performed in cells at logarithmic phase of growth.

### Determination of cytotoxicity

The viability of CHO, MCF-7, and MDA-MB 231 cells was determined by measuring the reduction of soluble MTT [3-(4,5-dimethylthiazol-2-yl)-2,5-diphenyltetrazolium bromide] to water insoluble formazan. The cells were seeded on 96-well plates at a density of 1 × 10^4^ cells per well and grown at 37°C in a 5% CO_2_ atmosphere. Following 24 h, cells were incubated with medium containing derivatives 1072, 1073, 1079, and commercial AZT (Sigma Aldrich—Missouri, USA) at various concentrations (0.1, 1, 10, 50, and 100 μM) for 4, 24, 48, and 72 h at 37°C. After these periods cells were washed twice with phosphate-buffered saline (PBS; Gibco, New York, USA); 5 mg/mL of MTT solution was added to each well and cells were incubated for 3 h at 37°C in 5% CO_2_. The medium was removed and then 200 μL of DMSO was added to each well, for solubilization of formazan crystals using a shaker for 20 min at 100 × g. The absorbance of each well was read on a microplate reader at a wavelength of 492 nm. The inhibition (%) of cell proliferation was determined as follows: growth inhibition rate (%) = [1 – (Abs_492_ treated cells/Abs_492_ control cells)] × 100. Results were expressed as media ± SD of three independent experiments performed in triplicate.

### Assessment of cell viability by live/dead assay

The LIVE/DEAD cell viability assay (Invitrogen®, Carlsbad, USA) was conducted following the manufacturer's instructions. Cells were cultured and incubated with the AZT derivatives as described above. Live cells were analyzed by green fluorescent light emission (488 nm), resulted from calcein uptake. Permeable membrane of dead cells allows diffusion of ethidium bromide homodimer and its binding to DNA, which was detected by the red fluorescent signal (546 nm). The results were analyzed in a Olympus IX71 fluorescence microscope (Olympus Optical Co., Tokyo, Japan) by multicolor imaging using a digital camera (Olympus, Tokyo, Japan). The recorded images were analyzed using Cell∧F software (Cell∧F, Olympus, Tokyo, Japan). The data were expressed as the mean ± SEM of percentage of dead cell, based 3 different fields of view, with 100 cells per field.

### Measurement of apoptosis by Annexin V staining

CHO, MCF-7, and MDA-MB 231 cells were seeded on 6-well plates at a density of 1 × 10^5^ cells per well. Twenty-four hours later, cells were exposed to 50 or 100 μM of AZT, 1072, 1073, or 1079. After 48 h of incubation, cells were trypsinized, centrifuged and washed in phosphate-buffered saline. The viable cell number in each well was counted using the Guava Via Count Assay (Merck, Darmstadt, Germany). Apoptosis was assessed using the Guava Nexin kit and the Guava TUNEL assay (Merck, Darmstadt, Germany).

### Analysis of gene expression by quantitative real-time PCR

To evaluate the expression profile of apoptotic genes, total RNA was extracted and cDNA was synthesized as previously described (20). Cells were seeded on 6-well plates at a density of 1 × 10^5^ cells per well and grown at 37°C in a 5% CO_2_ atmosphere. Cells were then incubated for 48 h with 50 or 100 μM of AZT, 1072, 1073, or 1079. After the incubation period, RNA extraction was performed using TRIzol® Reagent (Invitrogen®, Carlsbad, USA) followed by treatment with DNAse using a DNA-free kit (Ambion®, Carlsbad, USA). The cDNA synthesis was performed with an input of 2 μg of RNA using High Capacity cDNA Reverse Transcription kit (Applied Biosystems®, Carlsbad, USA). All steps described were performed according to the manufacturer's protocol. Real-Time PCR reactions were performed on a Stratagene® Mx3005P® Real-Time PCR System (Agilent Technologies, California, USA) using SYBR® Green PCR Master Mix (Applied Biosystems™, Massachusetts, USA) and primers described in Table 1. Validation experiments were previously conducted to ensure that efficiencies of all primer pairs were equivalent. Amplification was carried out using the following cycling conditions: 95°C for 2 min, 40 cycles of at 95°C for 15 s, and 60°C for 60 s. The melting curves were analyzed after amplification cycles at a linear temperature transition rate of 0.1°C/s from 55 to 95°C. Variations on gene expression were calculated using the 2^−ΔΔ*Ct*^ method (21).

**Table 1:** Primer sequences used in this study.

**Primers**	**Sequence 5′ → 3′**
*p53* For	AGCGAGCACTGCCCAACA
*p53* Rev	CACGCCCACGGATCTGAA
*Bcl-2* For	GTGTGGAGAGCGTCAACC
*Bcl-2* Rev	CTTCAGAGACAGCCAGGAG
*Bax* For	ATGCGTCCACCAAGAAGC
*Bax* Rev	ACGGCGGCAATCATCCTC
*Casp3* For	CAGTGGAGGCCGACTTCTTG
*Casp3* Rev	TGGCACAAAGCGACTGGAT
*Casp8* For	GGATGGCCACTGTGAATAACTG
*Casp8* Rev	TCGAGGACATCGCTCTCTCA
*Casp9* For	CCAGAGATTCGCAAACCAGAGG
*Casp9* Rev	GAGCACCGACATCACCAAATCC
*GAPDH* For	GGATTTGGTCGTATTGGG
*GAPDH* Rev	TCGCTCCTGGAAGATGG

### Data analysis

Data sets from MTT assay were analyzed using factorial ANOVA followed by a Tukey test for multiple comparisons. Three factors were considered: compound used (four levels), treatment time (four levels), concentrations (two levels). Data sets from Live/Dead, Annexin V, and real-time PCR were analyzed using a two-way ANOVA followed by a Tukey test for multiple comparisons. Two factors were considered: compound used (four levels), concentrations (two levels). Significance was considered at *P* < 0.05 in all analyses. All data were expressed as mean ± SEM.

## Results

### Determination of cytotoxicity

**Figure 2:**
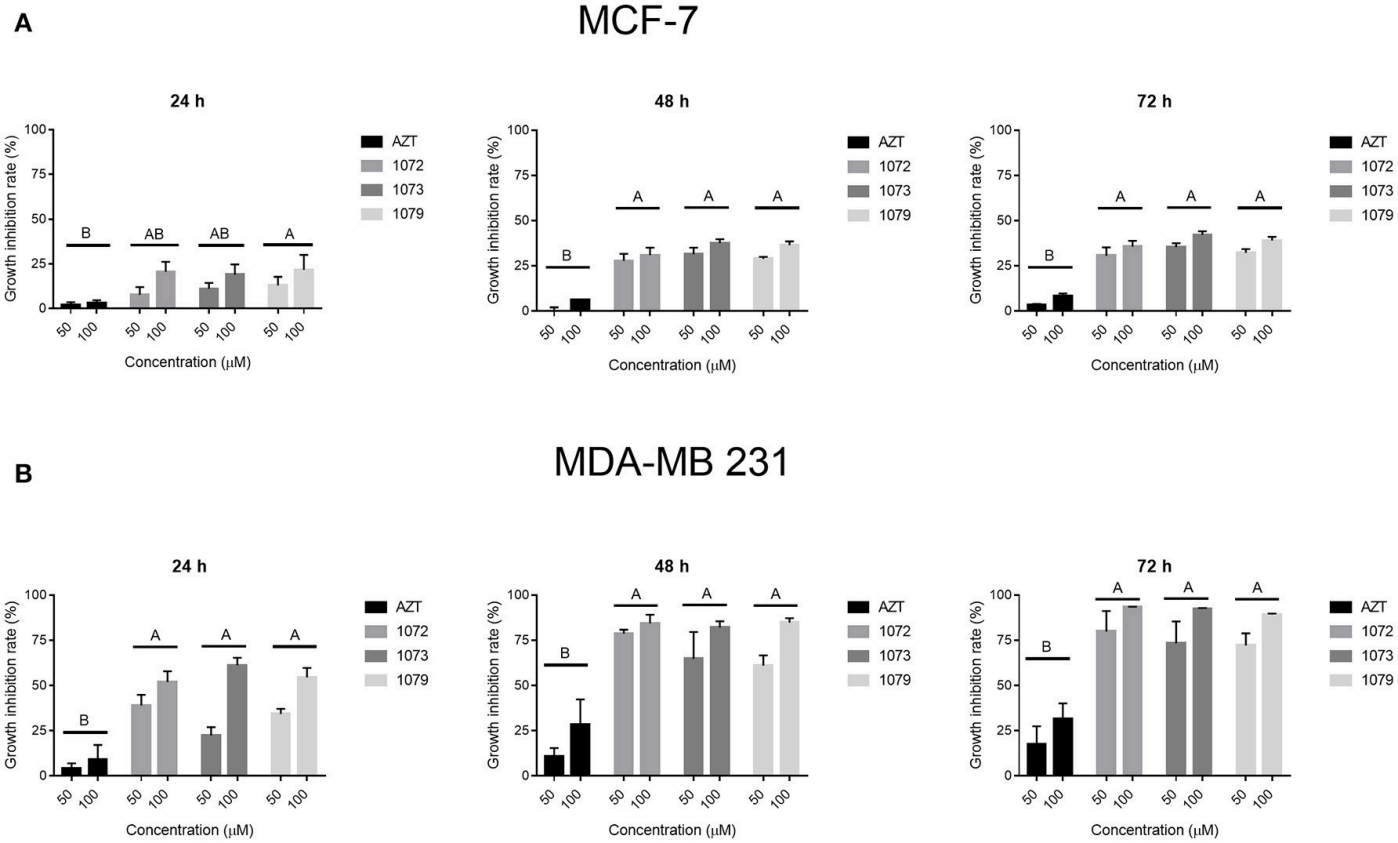
Effect of AZT and derivatives in cell proliferation. The cell lines **(A)** MCF-7 and **(B)** MDA-MB 231 were treated with AZT and derivatives (1072, 1073, and 1079) at concentrations of 50 and 100 μM in times of 24, 48, and 72 h. Cytotoxicity was assessed by MTT assay. Data are expressed as mean ± SEM from three independent experiments, performed in triplicate. Different letters above the horizontal lines indicate that there are signifcant differences among treatments at a *P* < 0.05.

The results from MTT assay (Figure 2) showed that incubation with AZT by 4, 24, 48, or 72 h did not induce cytotoxicity on CHO, MCF-7, and MDA-MB 231 cells. However, the derivatives 1072, 1073, and 1079 showed a selective decrease in cell proliferation, showing superior results in tumor cell line MDA-MB 231, with inhibition rates of 90% in concentrations of 50 and 100 μM in 48 and 72 h (Figure 2B). The tumor cell line MCF-7 showed intermediate results, with inhibition rate of 35% (Figure 2A). The control cell line (CHO) had lower rates of growth inhibition in the same concentrations and times in relation to tumor cell lines. The concentrations of 0.1, 1, and 10 μM showed no significant inhibition rates (data not shown). All compounds tested showed a decrease in cellular viability *in vitro* in a time-dose-dependent manner.

### AZT derivatives alter morphology of MDA-MB 231 cells

**Figure 3:**
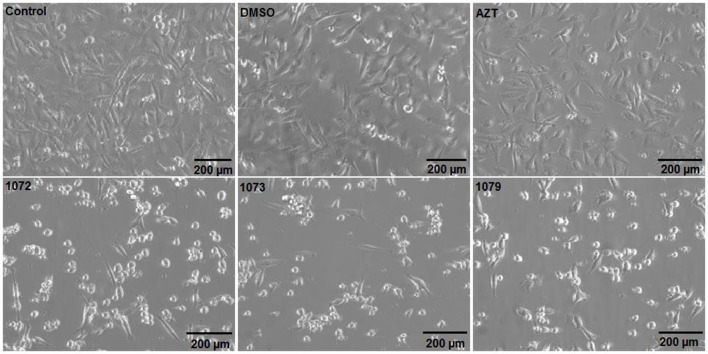
Morphological analyzes after treatment with AZT or derivatives. The tumor cell line MDA-MB 231 was incubated without treatment (control) or with 50 μM of AZT or derivatives (1072, 1073, and 1079) during 48 h. Cells treated with AZT derivatives showed morphology similar to apoptotic cells, with cell shrinkage and lose of cell-cell contact.

After treatment with the compounds 1072, 1073, and 1079 in a concentration of 50 μM during 48 h, MDA-MB 231 cell line showed apoptotic morphology, characterized by loss of attachment to other cells and extracellular matrix as well as rounding up. Cells incubated with AZT showed morphology similar to the non-treated control (Figure 3).

### AZT derivatives reduce cell viability

**Figure 4:**
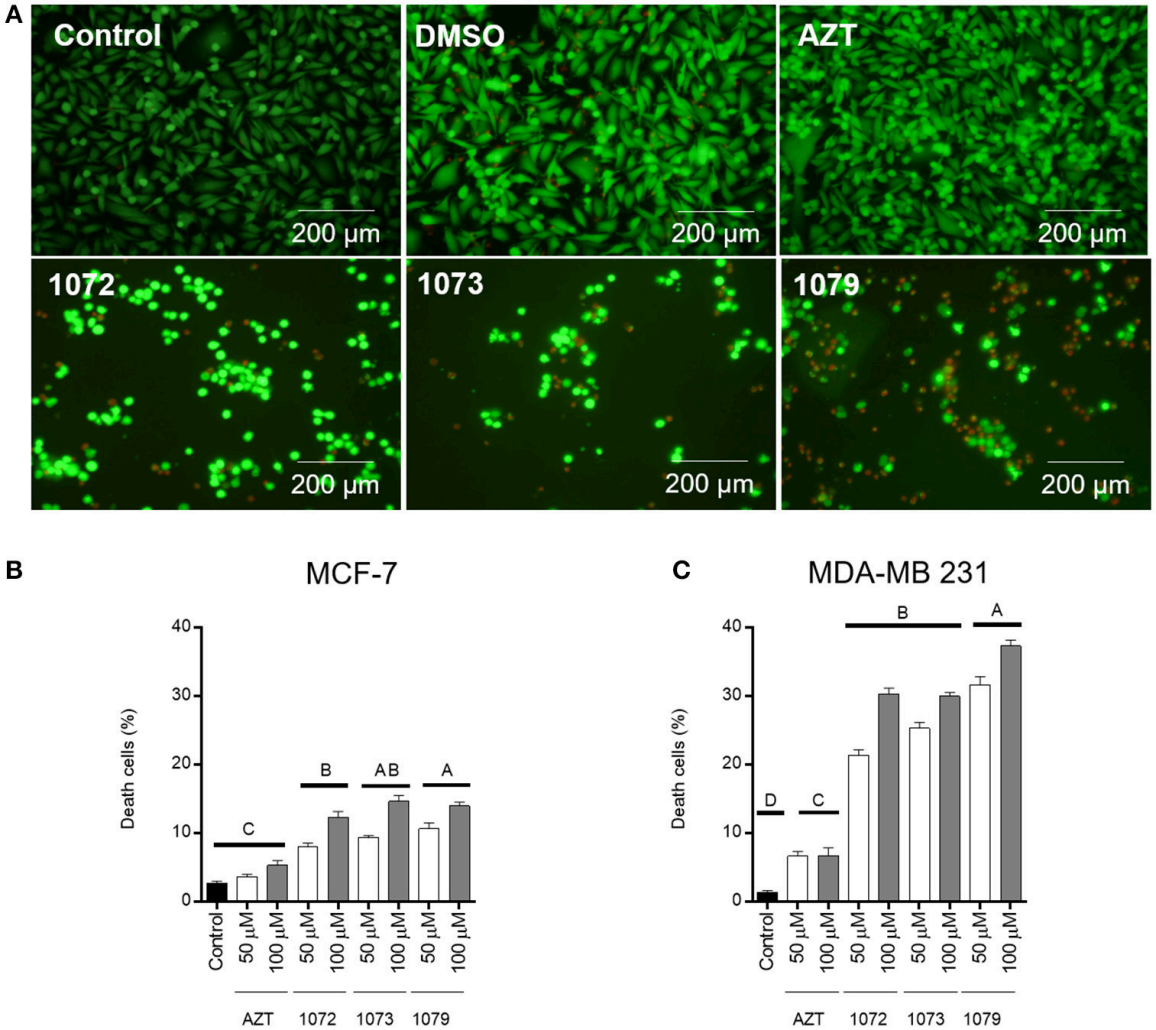
LIVE/DEAD cell viability assay. Representative figures of MDA-MB 231 incubated without treatment (control) or with 50 μM of AZT or derivatives (1072, 1073, and 1079) after 48 h **(A)**. Live cells are shown in green and dead cells are shown in red. The graphic shows the mean ± SEM of three different areas of the plate of MCF-7 **(B)** and MDA-MB 231 **(C)** cells. Different letters above the horizontal lines indicate that there are significant differences among treatments at a *P* < 0.05.

LIVE/DEAD, a two-color fluorescence assay, was conducted to evaluate cell viability after treatment with AZT derivatives. An increase in cell death (red fluorescence) after treatment with AZT derivatives was observed in line cell MDA-MB 231 when compared to control group (Figure 4). The reduction in cell number can be clearly observed in the tumor cell line MDA-MB 231, with a cell death rate of 35% (Figure 4C), while <15% was observed for MCF-7 cells (Figure 4B). There was a significant difference between the concentrations of 50 and 100 μM in compound derivatives (*P* < 0.05). DMSO vehicle had 5% of cell death, the same rate found for the control group (*P* > 0.05).

### Apoptosis analysis

**Figure 5:**
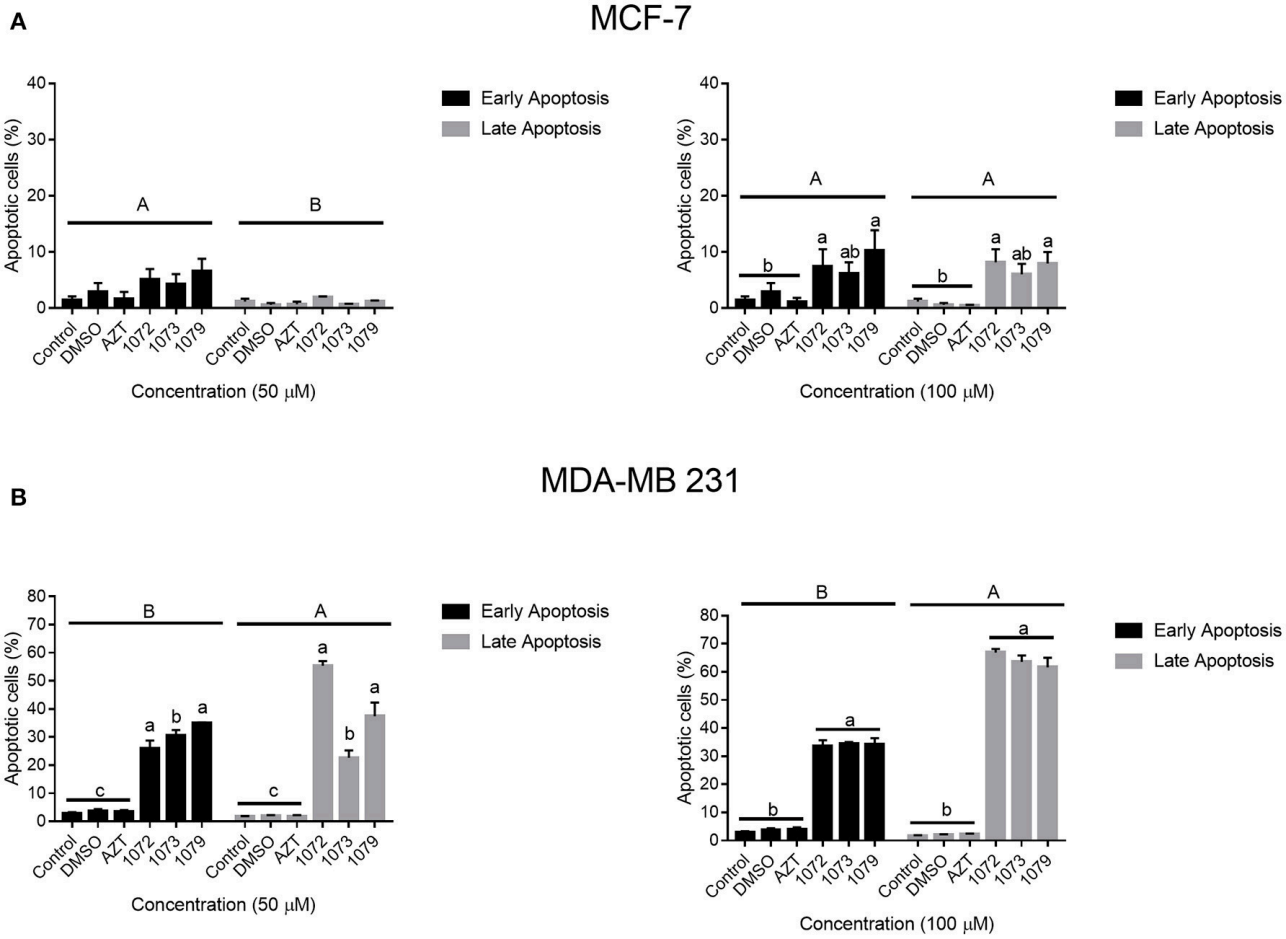
Induction of apoptosis by AZT and derivatives. MCF-7 and MDA-MB 231 cells treated with AZT and derivatives were examined for apoptosis by 7-AAD and Annexin V-PE staining. The graph shows the percentage of cells in early apoptosis (marked only with Annexin V-PE) and late apoptosis or dead (marked with V-PE e 7-AAD). Significant differences were considered at *P* < 0.05. Different capital letters indicate significant differences between stages of apoptosis. Different lowercase indicate significant differences between different treatments.

**Figure 6:**
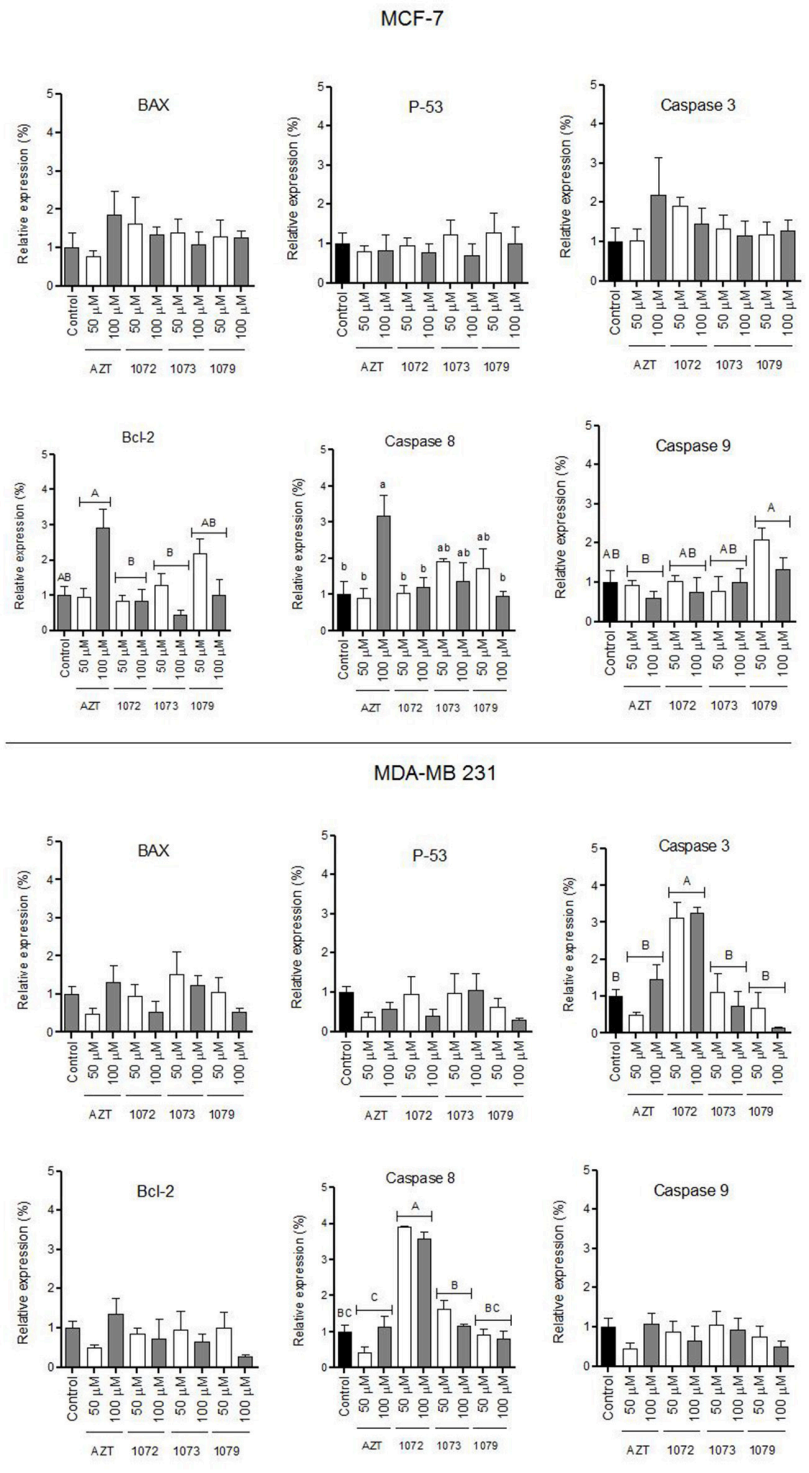
Gene expression profile. **(A)** MCF-7 and **(B)** MDA-MB 231 lines were treated with AZT and derivatives (in 1072, 1073, and 1079) at concentrations of 50 and 100 μM during 48 h. RNA and cDNA was extracted was synthesized. Relative expression data demonstrated a significant increase of caspase 3 and caspase 8 expression levels, in MDA-MB 231 cell line treated with the compound in 1072. Different letters above the horizontal lines indicate significant differences among compounds. For caspase 8 different letters indicate significant differences among the bars. Significant differences were considered at *P* < 0.05.

In order to analyze the induction of apoptosis by AZT, 1072, 1073, and 1079 in CHO, MCF-7 and MDA-MB 231 cells, we performed flow cytometry with annexin V-PE/7-AAD staining (Figure 5). Concentrations of 50 and 100 μM promoted significant different rates of late apoptosis in MCF-7 cell line (*P* < 0.05). The derivatives 1072, 1073, and 1079 induced rates of late apoptosis statistically different (*P* < 0.05) from control group in MDA-MB 231 cell line (Figure 6B). We also observed differences between the concentrations of 50 and 100 μM (*P* < 0.05). Compound 1072 at a concentration of 50 μM had a satisfactory response in the induction of apoptosis compared with the concentration of 100 μM. Treatment with AZT showed no difference compared to the control and DMSO (*P* > 0.05) vehicle.

### Analysis of gene expression

The expression levels of pro and anti-apoptotic genes (*Bax, Bcl-2, caspase3, caspase 8, caspase 9*, and *p53*) in MCF-7 and MDA-MB 231 cells were evaluated by qRT-PCR (Figure 6). Figure 6A shows the levels of expression in MCF-7 line. Figure 6B shows the levels of expression in MDA-MB 231 cell line, in which we observed significant difference compared to control for caspase 3 and 8 genes after treatment with derivative 1072 (*P* < 0.05). No difference (*P* > 0.05) was observed for the other genes evaluated (*Bax, Bcl-2, p53*, and *caspase 9*).

## Discussion

Several studies have demonstrated that AZT has antitumor activity and interesting biological properties (22–26). Chalcogenides compounds, such as selenium, were identified as chemopreventive agents that induce apoptosis in experimental models *in vitro* (18). Selenium compounds have shown anti-cancer effects especially based on production of reactive species of oxygen (ROS) and chromatin modification (27). Induction of apoptosis mediated by the combination of AZT with elements chalcogenides is considered a promising strategy for chemopreventive agents (9, 10). In this context, the present study demonstrated the effect of AZT and derivatives in breast tumor cell lines.

The breast tumor cell lines MCF-7 and MDA-MB 231 exhibit important differences regarding the presence of receptors for estrogen and progesterone, as well as for the human epidermal growth factor receptor 2 (HER2) (28). Furthermore, these tumor cell lines differ in the degree of malignancy (28–30). It is known that tumors that are positive for hormone receptors have a better prognosis (30), while triple negative tumors are more aggressive, resulting in lower treatment options (31–33). The MCF-7 line is positive for estrogen and progesterone receptors and negative for HER2 protein, while MDA-MB 231 line is triple negative for these receptors (28).

The MTT assay was conducted to detect cytotoxic effects of AZT and its derivatives (1072, 1073, and 1079) on MCF-7 and MDA-MB 231 cell lines. AZT showed a low rate of growth inhibition in all strains tested, indicating its decreased efficacy at the concentrations evaluated. Similarly, low levels of inhibition were observed in the CHO cell line for all compounds tested. However, derivatives of AZT were able to significantly increase the rate of growth inhibition in tumor cell lines, with the most important results in MDA-MB 231, an invasive cell line. These findings suggest a selective action of the AZT compounds on tumor cell lines, which may be supported by the differential expression of receptors in these cells, involving multiple intracellular signaling pathways (34, 35). Selenium-based molecules have shown efficacy and high selectivity as chemotherapeutic compounds (27). The higher rates of growth inhibition in the more aggressive line MDA-MB 231, highlight the selective potential of the compounds tested in this study. The Live/dead assay confirmed the results obtained by MTT. Cells treated with AZT showed a low percentage of viability, similar to what was found in control and drug vehicle groups. These data show the importance of the modifications in AZT derivative compounds in order to obtain a significant antiproliferative effect (14, 22).

Therapeutic targets involve intracellular signaling networks, leading to changes in gene expression, cell energetics, immune modulation, arrest of the cell cycle, and/or apoptosis (2). Apoptosis is the process of programmed cell death, one of the targets of antitumor therapies (36). Through Annexin-V/7-AAD analysis, we demonstrated that the derivatives of AZT induced apoptosis. An increase in both early and late stages of apoptosis was observed in tumor cell lines MCF-7 and MDA-MB 231, with the more significant apoptosis rates in the line MDA-MB 231. Annexin V binds to cells that present externalized phosphatidylserine, an indicative that cells are entering in apoptosis (37).

One family of proteases, the caspases, has long been considered the main performer of all programmed cell death (38). When recruited by the extrinsic pathway, which is initiated by activation of cell surface death receptors, the caspase 8 activates caspase 3 (36). The activation of caspase 3 promotes the degradation of cellular proteins and chromosomal DNA, leading to loss of cell integrity (39). Our data show that the derivative 1072 of AZT increased the expression of transcripts of the genes caspase 3 and 8 in the line MDA-MB 231 compared to control, suggesting that the extrinsic pathway of apoptosis was activated after treatment with this compound. These results are in accordance with other studies that analyzed the antitumor action of compounds containing selenium in its formulation (19, 40, 41).

The antiproliferative effects were better observed at longer times of exposition (48–72 h), suggesting a mechanism of action mediated by a slow gene expression regulation. Although the more promising results have been obtained with the highest concentrations of AZT derivatives (50–100 μM), no cytotoxic effect was observed in non-tumor cells at these doses. Our study showed a cytotoxic and selective effect of AZT derivatives in different breast tumor cell lines, indicating their potential as chemotherapeutic agents. Moreover, the efficacy of these compounds in MDA-MB-231 cell line highlights their ability to overcome limitations in treatment of triple negative breast cancer.

In conclusion, our results indicate that derivatives of AZT promote cytotoxicity *in vitro* in the line of triple negative breast cancer, probably through activation of the extrinsic pathway of apoptosis as demonstrated for 1072 derivative. These compounds containing selenium in its formulation are potential therapeutic agents for breast cancer.

## Author contributions

MW, IO, DS, OR, TC, and FS conceived and designed the experiments. MW, ES, PdL, and HT performed the experiments. MW, VC, TC, and FS analyzed the data. MW, ES, TO, TC, and FS wrote the paper. MW, ES, TO, PdL, HT, VC, IO, DS, OR, TC, and FS final approval of the version to be submitted.

### Conflict of interest statement

The authors declare that the research was conducted in the absence of any commercial or financial relationships that could be construed as a potential conflict of interest.
